# Adapt or perish? Assessing the recent shift in the European research funding arena from ‘ELSA’ to ‘RRI’

**DOI:** 10.1186/s40504-014-0011-x

**Published:** 2014-05-14

**Authors:** Hub Zwart, Laurens Landeweerd, Arjan van Rooij

**Affiliations:** Department of Philosophy and Science Studies, Faculty of Science, Institute for Science, Innovation and Society (ISIS), Radboud University Nijmegen, P.O. box 9010, 6500 GL Nijmegen, the Netherlands

## Abstract

Two decades ago, in 1994, in the context of the 4^th^ EU Framework Programme, ELSA was introduced as a label for developing and funding research into the *ethical, legal and social aspects* of emerging sciences and technologies. Currently, particularly in the context of EU funding initiatives such as Horizon2020, a new label has been forged, namely *Responsible Research and Innovation* (RRI). What is implied in this metonymy, this semantic shift? What is so new about RRI in comparison to ELSA? First of all, for both labels, the *signifier* (S) was introduced in a top-down manner, well before the concept that was signified by it (s) had acquired a clear and stable profile. In other words, the signifier preceded (and helped or helps to shape) the research strategies actually covered by these labels (the precedence of the signifier over the signified: S/s). Moreover, the newness of RRI does not reside in its interactive and anticipatory orientation, as is suggested by authors who introduced the term, but rather in its emphases on social-economic impacts (valorisation, employment and competitiveness).

## Introduction

Science and technology are powerful engines of change, and generate heated controversies as well as high expectations. In response to this, sensitivity to societal issues, particularly within the life sciences, has increased over the years and has become a standard feature of funding programs. No less controversial than the normative and/or societal issues themselves, however, are the ways in which they are framed and the extent to which they become enlisted or delisted from various agenda’s.

In Europe, two decades ago, in the context of the 4^th^ EU Framework Programme (launched in 1994), a new label was introduced to frame societal issues and to finance research, stakeholder dialogues, education and other activities to address them, namely ELSA: an acronym that stands for *ethical, legal and social aspects* of emerging sciences and technologies. The key signifier is the letter A, inserted at the end in order to distinguish it from ELSI, which refers to research on ethical, legal and social *implications* of emerging life sciences, notably human genomics, - a program funded by NIH/NHGRI in the U.S. (as part of the human genome sequencing initiative). Since then, the ELSA label has been adopted by other funding initiatives as well, notably in European partner countries, for instance the ELSA program of the Research Council of Norway and ELSAGEN (a collaboration of research funding agencies in Germany, Austria and Finland) (Chadwick and Zwart 2013). Although the acronym ELSA was coined as a top-down funding mechanism, by funding agencies rather than by the research communities themselves, it nonetheless managed to evolve into a recognisable approach (Zwart and Nelis 2009). In other words, rather than being an ‘empty signifier’, ELSA actually came to *signify* something, namely a particular research practice, with researchers using the opportunities ELSA programs offered (for instance in terms of relative proximity to large-scale life sciences research consortia) to strengthen the visibility and impact of their work. As Stegmaier (2009) phrases it, ELSA programs tend to fund activities that are geographically and organizationally close to the life sciences research programs with which they interact, serving as public and academic forums for addressing urgent societal issues arising in this context. The question whether and to what extent the research groups involved had really ‘internalised’ the label (seeing themselves as representatives of the ELSA-approach) has been addressed at various occasions, for instance by the *Centre for Society and Genomics* (funded by the *Netherlands Genomics Initiative*), via a workshop in September 2008 involving ELSA researchers from various countries (United Kingdom, Netherlands, Germany and Switzerland). In his report of this event, Peter Stegmaier (2009) stresses the diverse and often experimental nature of the various activities carried out under the ELSA heading. Nonetheless, a certain amount of “convergence” can be discerned as well, although “the question of what is bottom-up—initiated by genomics or social researchers—and what is top-down—initiated by management or government—remains hard to answer. Initiatives come from all sides and the top-down programmes, in particular, are brought to life by researchers from the bottom-up in ways that governance could never have foreseen” (p. 115).

Currently, and particularly in the context of recent EU funding initiatives (notably Horizon 2020), we witness a new initiative in the labelling arena, namely a shift from ELSA towards *Responsible Research and Innovation*, also known as RRI (Von Schomberg 2011, 2012, 2013; Owen et al. 2012). It is important to notice that, once again, this neologism is not introduced by the research field itself (‘bottom-up’), but rather by science policy makers and funding agencies (notably within the European Commission) in a top-down manner, although the term seems to strike a cord and concurs with concomitant funding stratagems on the national level as well, for instance in the Netherlands, where RRI is known as MVI (‘Maatschappelijk Verantwoord Innoveren’). Moreover, quite recently, a new journal, the *Journal for Responsible Innovation* has been launched by Taylor and Francis: an indicator of the readiness within the field (or at least among certain fractions within the field) to embrace/applaud (or at least adapt to) this relabeling move.

In other words, we are faced with a series of terminological shifts known as *metonymy*, to use the linguistic term for this phenomenon. A particular concept (in this case: ELSA) is no longer referred to by its usual name, but suddenly figures under a slightly different heading, one more or less associated it. Whereas ELSA always (to some extent at least) has been about safeguarding socially robust forms of fair and responsible innovation, some specific features are now more explicitly placed up front to re-frame and re-focus the approach as such. Notably, economic valorisation is given more prominence. There clearly is congruency, but a shift of focus can be discerned as well. Similar patterns of re-labelling tend to arise within life science domains themselves, of course, where labels such as ‘genetics’ came to be refashioned as ‘genomics’ (and its various derivatives), in order to reset the general focus of the field, while currently genomics is giving way to systems biology, synthetic biology, personalised medicine and other ‘post-genomics’ headings (not completely unlike genomics, but with slightly different emphases).

As Freud (1900/1942) and Lacan (1966) have pointed out, however, metonymy (or ‘displacement’, *Verschiebung* in German) hardly ever represents a ‘neutral’ gesture. Quite the contrary, from a psychoanalytic viewpoint, metonymy is closely connected with (efforts to adapt to) strategies of censorship. In other words, it is by adopting this new label that the field in question hopes to be recognised as ‘eligible for funding’ by authoritative bodies such as funding agencies. The ‘latent’ research question (researcher-driven, as it were) has to be reframed in such a manner that it may fall under the heading of RRI, so as to ensure that obstacles connected with criticism of (or with biases towards) previous approaches (such as ‘bioethics’ or ‘ELSA’) may be circumvented. This type of semantic flexibility (perhaps even masquerade) comes with a price, however. It often means that some types of questions will become more important (or more difficult to ask) than others.

Phrased in Lacanian terms, the question arises what this game of signifiers (notably the recent shift from ELSA to RRI) implies on the level of the ‘subjects’, i.e. the researchers and research groups involved. On the one hand, they depend on key signifiers to develop a more or less stable identity. On the other hand, with every new signifier appearing on the horizon, something will be gained and something will be lost. A recognisable label furnishes the subject with a discursive ‘life-line’, as it were. Without a credible label, the subject is left to his/her own devices and remains fragile and ‘divided’ (*$*), so that access to academic discourse and funding will be questionable and limited. The previous shift (on the level of signifiers) from ELSI (S_1_) to ELSA (S_2_) allowed the researchers involved to cleanse themselves of certain undesirable connotations connected with the ELSI label (such as the reproach of being too supportive of genomics research as such) and to develop a new identity (involving more opportunities for taking a critical stance, for instance). The question now is what is gained and what is lost with the current shift from ELSA to RRI.

As we will argue in this paper, whereas ELSA (S_2_) managed to develop a more or less recognisable profile over the years, allowing researchers to acquire a fairly established identity under this heading, the conceptualisation of RRI (S_3_) is still rather open-ended, for the time being at least. And yet it easily seems to brush the ELSA approach aside, putting the subject in a position that is both highly vulnerable and dense with opportunities. We will also argue, moreover, that this metonymy/displacement from ELSA to RRI must be regarded as a *symptom* reflecting a more fundamental shift/tension within the discourse and practice of research concerning the societal dimensions of life sciences innovations as such. Notably, within the RRI format, much more emphasis is now given to collaboration with industry and to potential socio-economic benefits of scientific and technological change. This not only pushes researchers into close proximity to their private-public ‘objects’ of research, but may also infect them with the aims and ideologies involved, such as: innovation, creating jobs and similar tangible socio-economic impacts. In other words, under the heading of RRI, ELSA may have lost (what was still left of its) innocence.

Rather than merely signalling these shifts, from a safe distance as it were, we would like to reflect on them from a position of proximity and involvement, as ‘engaged scholars’ so to speak. How to address the challenges involved, how to position ourselves vis-à-vis the developments outlined above? Basically, we will argue that a shift of signifiers is not an innocent game, and that losses may be involved. In other words, ELSA should not be brushed aside too quickly. Rather, (the conceptualisation of) RRI should build on the strengths of and past experiences of ELSA practices. Nonetheless, we recognise the importance of an open mind when it comes to acknowledging ELSA’s weaknesses as well. We should be willing to at least consider new pathways for governance of research and innovation in the current era. At the same time, a reflection on the latent tensions reflected by this metonymy will make us more alert to some of the ideological threats and risks involved in working along the lines of the RRI concept. Thus, we will develop a preliminary assessment of RRI against the backdrop of a retrospect on ELSA, while fleshing out a number of theoretical and practical implications in this shift in grammar.

As to methodology, the focus of our paper is on (ELSA and RRI) *discourse*, rather than on *practice*, on conceptual analysis, rather than on empirical research. In other words, rather than discussing how ELSA/RRI is actually done, our paper addresses the way in which these basic approaches are fleshed out in primary and secondary sources. The focus is on the concepts, objectives and ideals, rather than on the work actually conducted under these headings. We will assess ELSA, RRI and their predecessors in chronological order. The composition of the paper, therefore, is as follows:

## Part I: ELSA - and its challenges: a retrospect

### Predecessors of ELSA

The assessment of societal dimensions of science and technology often involves a variety of ‘third’ parties besides scientists. In the past decades, these have included legal and ethical experts, NGO’s, policy makers and a variety of ‘publics’. In recent years, collaborative interactions with relevant stakeholder representatives were increasingly seen as a promising alternative to the ‘expert’ model. The various approaches emerging in this context all have their strengths and weaknesses, so it seems. Notably, finding a proper balance between embedded activity (‘proximity’) and autonomy (which is more easily maintained when working from a distance) is not self-evident. It is important, however, to see ELSI, ELSA and RRI (and the various discussions they raise) as efforts to respond to criticisms of previous approaches.

Around 1990, existing philosophical, bioethical and Technology Assessment (TA) approaches to science and technology were increasingly seen as insufficient. Whereas philosophy was often regarded as a marginal, ivory-tower phenomenon, bio-ethics was seen by many as a mere service provider for scientists, while TA was considered to be a fairly technical field. Public engagement on the other hand seemed too open to gut-feelings, untested emotions and single issue politics. Against this backdrop, ELSA developed, using combinations of these approaches (Zwart 2012). Before turning to ELSA proper, we will therefore briefly assess a number of complaints regarding these more ‘traditional’, pre-ELSA forms of research that (notably after 2000) gradually become ‘infected’ with the new model. These brief historical portrayals are not meant to be exhaustive (more comprehensive reviews are available elsewhere), but merely help to set the stage.

#### Philosophy

As was already mentioned, one key issue in the reflection on science and technology is the issue of ‘distance versus proximity’. Philosophers are often seen as preferring a position of detachment and distance towards science/technology as targets of research. Nonetheless, the plea for proximity to science has had various advocates in philosophy long before ELSA came about. One such voice has been the influential French philosopher Michel Serres (1972), who already in the 1970s argued that we live in an era of disruptive change, in science as well as in politics. As an important symptom of the transition, he mentioned the emergence of large-scale, high-tech, trans-disciplinary research fields such as molecular biology. Serres pointed out that philosophy runs the risk of losing track of these high-pace developments, thereby becoming outdated and irrelevant. Yet, according to Serres, philosophers could still play a pivotal role, provided that they acquaint themselves with these newly emerging worlds and ‘oceans’ of knowledge from a position of close proximity. To do so, they would have to enter the capillaries and tissues of techno-science and really become embedded scholars, addressing the various philosophical issues raised by these developments in close interaction with the scientists who are active within these fields. One important objective for philosophy is what Serres refers to as “conceptual epidemiology”: analysing and assessing how techniques, vocabularies, research practices, forms of information and metaphors spread through research fields worldwide, infecting and inflaming the tissues of the broader societal life-world as well. This task, we believe, is as relevant now as it was in the 1970s.

#### Bioethics: the professional and the participatory turn

One could argue that professional bioethics, as it emerged during the 1980s, tried to steer a kind of middle-course between proximity and distance (as well as between criticism and support) of (predominantly biomedical) research practices. In a well-known article entitled “How medicine saved the life of ethics”, Stephen Toulmin (1982) argued that the involvement of professional ethicists in addressing moral dilemmas, emerging in contemporary medicine and health care, had allowed the field to revivify itself. Ethics suddenly evolved from a dull, self-centred and fairly marginal academic sub-discipline into a thriving, interdisciplinary research area. Whereas in the 1970s critical monographs written by single authors played a dominant role (with Ivan Illich’s *Medical Nemesis* (1977) as the classical example), the 1980s and 1990s witnessed a process of professionalisation, exemplified not only by the emergence of ethics committees in academic hospitals, but also for instance by the launch of the journal *Bioethics* in 1986 and, more generally, by the rise of the ethical expert model. Rather than fleshing out a broad and comprehensive view of the role of medicine in contemporary culture, as Illich and others had done, professional ethicists opted for a consensus model, which entailed a focus on case studies, concrete dilemmas and principles. On the negative side, it resulted in a rather drastic delisting of the more substantial issues from the agenda of bioethical discourse. Virtually all items pertaining to ‘metaphysical issues’ (What is nature? What is life? What is embodiment?), ‘world-views’ (either secular or religiously inspired), or to the interwoveness of science, technology and society at large, tended to be regarded as being beyond the scope of ethical deliberation. Bioethics increasingly retreated to procedural aspects of ethical deliberation and to the process of weighing ‘self-determination’ versus ‘harm to others’, at the expense of broader content (Zwart 2001).

During the 1990s, ethics expertise was generally seen as an appropriate basis for advice to policy makers on ethical issues in science and technology, on national and European levels. In this capacity, it came to be institutionalised as a policy instrument rather than as an academic discipline (Tallacchini 2009). A good example of the prominence of ethics as a governance tool have been the evaluation processes for EU Framework Programme research applications. Over the years, ethical review became increasingly prominent. Proposals for research, once they were evaluated positively in terms of scientific quality, were assessed in terms of their ethical quality as well, by panels of ethics experts drawn from across the EU^a^. Bioethics came to be seen as a major source for assessment, regulation and justification. As such, bioethics evolved into a normative instrument for policy and legislation (Tallacchini 2009). Yet, it was especially this success of bioethics in terms of its institutionalised role which triggered a broad variety of concerns. Eckenwiler and Cohn (2007) notably voiced concerns with regard to the tension between bioethics as acting on behalf of morality, and bioethics as an institutionalised element *within* the field it purported to assess, conveying a ‘technocratic style’ of analysis. This gave rise to the criticism that bioethicists had become advocates and facilitators of science and technology rather than critical assessors of their societal impact.

Discontent with the professional bioethics model resulted in new developments in at least two directions, namely on the one hand the rise (during the 1990s) of the public participation paradigm, and on the other hand the coming into being of ELSA research, where bioethics was combined with empirical and participatory approaches. While public participation focussed on involving (future) stakeholders and various ‘publics’ (plural) in reflections on new technologies (Broerse 2011), ELSA research took a slightly different course. The focus was on increased collaboration and interaction between experts from various fields: notably between researchers from the humanities and social sciences on the one hand with life scientists and life science research consortia on the other, although in practice both approaches (public participation and ELSA) could be combined in various ways, notably in the context of research that involved focus-groups with scientists, social sciences and humanities experts and (future) stakeholders (Broerse 2011).

#### Technology assessment: the co-constructive turn

Technology assessment (TA) was the dominant approach in science governance before the 1990s. Traditional TA, however, was seen by many as a rather technical form of research, conducted by science and technology experts, often with a background in science or engineering. It pretended to be neutral, but nonetheless seemed to convey an instrumentalist vision on both society and technology, seeing technologies as tools that could enable us to solve certain problems, depending on the circumstances. Gradually, the idea emerged that the concept of expertise should be broadened and that societal expertise (represented by future users) should be involved in this process. An important exemplification of the participatory turn within TA is *Constructive Technology Assessment*, developed by Arie Rip and others (Robinson 2010, Krabbenborg 2013). In the context of CTA, the social scientists, instead of being ‘experts’ themselves, rather play the role of mediators, ‘bridging’ separate worlds by organising dedicated ‘bridging events’^b^.

CTA builds on the concept of ‘co-evolution’ between science and society that was put forward by ‘science and technology studies’ (STS) scholars, a branch of sociology focusing on the social shaping of emerging technoscience. Nowotny et al. for instance distinguished between ‘mode-1’ (disciplinary, predictive, linear) and ‘mode-2’ (context-driven, problem-focused, interdisciplinary) research, giving priority to the latter, which is oriented on integration of science and technology with society and vice versa. Similarly, Moor and Weckert (2003) state that ethics should already be integrated in science and technology processes in an early stage: the “ethics first”-approach. By this, they mean an approach to innovation in which ethical considerations (concerning both positive and negative societal impacts) are already taken into account in the design-phase of innovation trajectories (i.e. ‘upstream innovation’), rather than defining moral constraints for innovation in hindsight. Many of these approaches exemplify a move from *analysing* how science and technology are shaped by societal factors to actively *supporting* ways to integrate science and technology in society.

### The launch of ELSI

Against the backdrop of discontent with existing approaches, and building on concepts such as ‘mode 2’, ‘ethics first’ and ‘upstream innovation’, ELSI (in the U.S.) and ELSA (in Europe) came about. ELSI came first (in 1990), while ELSA (introduced in 1994) was partly modelled on ELSI, but at the same time aimed to move beyond some of the restrictions of the ELSI format.

ELSI was conceived in 1988 when James Watson, at the press conference announcing his appointment as director of the Human Genome Project (HGP), suddenly and unexpectedly declared that the ethical and social implications of genomics warranted a special effort and should be directly funded by NIH (Cook-Deegan 1994/1995). Like the HGP itself, the US ELSI program was formally established in 1990. Its mission was to anticipate and address the ethical, legal, and social implications of genetic and genomic research. From 3 up to 5% of the NHGRI Research budget was devoted to this type of work. Thus, NIH all of a sudden became the largest public funder of bioethics research world-wide. Over the years, various ELSI and/or ELSA programs have been developed, in Canada,^c^ Europe and the Far East (Chadwick and Zwart 2013).

In an issue of *Nature* published on November 9, 1989 (the day of the collapse of the Berlin Wall) an article entitled ‘Dealing with the data’ describes how HGP researchers were setting up a joint database where scientists could deposit their sequencing data^d^. Interestingly, this article was flanked by a second one entitled ‘Ethical matters’, containing a report of an international meeting outlining the societal hazards involved in human genome sequencing. This was a nice early example of the ELSI approach: genomics scientists on the one hand, experts from the social sciences and the humanities on the other, working side by side, as flanking endeavours, to create optimal conditions for an adequate embedding of genomics, notably of its flagship project, the HGP.

From the very outset, like some of its predecessors (such as bioethics), ELSI has triggered scepticism and criticism from various corners. In a paper entitled *What’s ELSI got to do with it?* Michael Yesley (2008) argues that, despite its unprecedented size, the ELSI program (“the richest bioethics program in history”, p. 4) failed to have noticeable impact. Moreover, it was dedicated to “advancing” rather than to critically assessing genomics/genetics research. Also, although ELSI “produced a large portfolio of academic literature primarily intended for, and communicated to, other academicians” (p. 2, p. 5), it had little or no influence on policy. According to Yesley, an advisory committee would have done a much better job.

From our perspective, the fairness and validity of Yesley’s analysis may be questioned. The juxtaposition of ‘academic research’ *versus* ‘policy relevance’, for instance, seems unconvincing. Quite the contrary, we would argue, for how can policy be informed/improved in the absence of solid evidence-based research? Moreover, his claim that ELSI (in order to “advance” the scientific research it studied) has *avoided* to address more critical issues such as “genetic reductionism”, “biological warfare” or the “priority” accorded to genomics/genetics in budget spending, is refuted by the actual ELSI output, which includes highly critical scholarly works, such as (to mention just one example) Lily Kay’s (highly influential) analysis of the entanglement of molecular genetics and the militarisation of post-war American science (Kay 2000). Often, such criticism has been brought forward against ELSI and ELSA in one and the same breath. Browsing through the tables of contents of typical ELSI/ELSA journals such as *New Genetics and Society* or *Life Sciences, Society & Policy*, shows that the accusation that ELSI (and/or ELSA) is only about streamlining implementation of new technologies does not hold. Nonetheless, ELSA programs tend to have a slightly different approach path (more bent on ‘academic criticism’) than the prototypical ELSI project, as will be more fully explained below.

### Birth of ELSA (or: the ELSI → ELSA shift)

As we have seen, the acronym ELSI (in the US) or ELSA (in Europe) refers to research and interaction activities that aim to anticipate and address ethical, legal and social *implications* (ELSI) or *aspects* (ELSA) of emerging life sciences, notably genomics. The shift in signifiers, from I (implications) to A (aspects) was generally seen as an effort to broaden the scope of the research (notably: to avoid the flawed linearity implied by ‘implications’ (Wickson et al. 2014)) and to launch a European alternative to the American counterpart. In the United Kingdom, this lead to a network of ELSA centres funded by the *Economic and Social Research Council* (the ESRC Genomics Network: EGN). In the Netherlands, the *Netherlands Genomics Initiative* (NGI) was established. 5% of its budget was spent on ELSA activities, on the one hand in the form of a research program issuing calls for researcher-driven, stand-alone projects (entitled: “The societal component of genomics research”) and, on the other hand, in the form of a *Centre for Society and Genomics*, established in 2004. CSG evolved into a large-scale centre for interactive research and communication, with approximately 50 research projects designed and conducted in collaboration with the other 15 centres of the NGI genomics network. A budget of 25 million € was available for research, communication and education. Besides the substantial amount of funding involved, ELSA Genomics research in the Netherlands provided challenging opportunities for other reasons as well. It was regarded as a test-bed for a new style of doing research, as well as for setting up a new type or organisation. As a national Research Centre, CSG developed a relatively open network of trans-university collaborations for developing and conducting an interactive research programme of substantial size. Indeed, in purely quantitative terms, ELSA could be regarded as the Social Sciences and Humanities version of ‘Big Science’. A key feature of the program consisted in proximity to (and collaboration with) prominent large-scale life sciences research programs. Virtually all research projects of the CSG program entailed collaborations or at least interactions with genomics research centres at various stages of the research trajectory (Zwart 2013). Through these and similar initiatives, an ELSA research community, described by Stegmaier (2009) and others (for instance: Wickson et al 2014), unfolded.

### Challenges of the ELSA approach

ELSA was forged to amplify the strengths and forego the weaknesses of the previous approaches discussed above, in response to the on-going quest for ways to institutionalise normative assessment of science and technology in European policy processes. Point of departure was the view that scientific expertise as such cannot sufficiently guarantee the societal justification of developing and introducing new technologies. Specific strategies need to be developed, first of all in order to forego future public resistance (such as in the case of the GMO debacle), but also because a large portion of scientific research is funded by public means. Therefore, society should have a say in how the money is invested, i.e. trans-mutated into knowledge (which, hopefully, and eventually, can be trans-mutated back into money once again, through a process which is now often referred to as ‘valorisation’). Moreover, society will also function as the future *consumer* of the scientific knowledge thus produced, and as the future *market* for new technological devices. Thus, it is highly relevant to be able to determine at a relatively early stage whether certain applications will be considered acceptable or usable or not, as public controversy will hinder innovation. Also, it is acknowledged that science and technology practices call for a broadening of the concept of responsibility. The internal moral codices of science should not merely involve issues of risk, safety and security, but should also include issues of social responsibility in a broader sense (such as: bringing about a more sustainable future). ELSA thus became the concept under which normative critique of science and technology was to be institutionalised. It held the two-way objective of critical assessment of on-going research on the one hand and of facilitating the future embedding of science (via co-constructive agenda-setting and policy advice, for instance) on the other.

Over the years, much like its predecessors, ELSA research has been confronted with a number of internal and external challenges, or even “pathologies”, as Wickson et al. (2014) have phrased it. To begin with, a problem of ELSA agenda-setting has been that research agendas of ELSA research were often pre-formatted by the scientific research programs they intended to study. The scientists had a say in the type of problems ELSA researchers were expected to address. This concern is of course reminiscent of the earlier discontent with regard to bio-ethics. Similar issues of pre-formatting and framing had played a role in embedded bio-ethics activities as well. Ethics advice committees, for example, were often seen as functioning within pre-defined procedures and aims: coming to consensus, finding solutions to tricky issues. This made them vulnerable to the accusation that they de-listed problematic items or ‘closed’ controversies over ‘deeper’ or ‘broader’ issues, as we have seen. Due to its proximity to science, and to large-scale funding initiatives for large-scale research programs, ELSA now became the target of similar concerns.

Also, and in relationship with a (real or perceived) dominance of ethics (notably medical ethics), ELSA (like its predecessor ELSI) has been accused of focussing on a limited set of issues, notably individual autonomy (and related items such as privacy) and harm or risk. Yet as science and technology, notably in the life sciences, included research in areas such as synthetic biology and nanotechnology as well, it tended to move far beyond the areas of health applications, involving issues such as bio-fuels, biomaterials, waste management and industrial bio-tech. Thus, whereas ELSA-expertise tended to remain associated with medical ethics, stressing issues of health risks and patient autonomy, other concerns and issues (naturalness, identity, global justice etc.) tended to be neglected. Overall, ELSA was often seen as an endeavour that focuses on the health domain rather than on agriculture, industry or the environment. In response to this, the ELSA community began to broaden its scope and to seek new partnerships with scientists.

In a reflection on the CSG program mentioned above, the following physiognomy of ELSA research was presented (Zwart and Nelis 2009). First of all, the authors emphasise that ELSA must *not* be seen as a new ‘discipline’ or area of research. Rather, they argue that the ELSA acronym basically refers to a particular *style* of doing research, to a basic methodological *attitude*, one that may apply to all the disciplines involved (bio-ethics, philosophy of science, STS, TA, science communication, etc.). According to the authors, ELSA research involves:

- Proximity to life science research
- An anticipatory, forward-looking approach; a focus on the agenda-setting and design stages of innovation trajectories, rather than on the product stage
- Interaction with a broad range of societal stakeholders (media, policy, NGO, industry) as integral part of the research
- Interdisciplinarity: ELSA research as a converging field involving a broad range of disciplines (philosophy of science, bioethics, social science, TA, STS, innovation studies, science communication etc.)
- A focus on micro-analysis (“case studies”) rather than on macro analysis (socio-economic studies)
- A tendency to draw on a wide variety of sources: from academic philosophy via policy reports up to media coverage of public debates and genres of the imagination (novels, plays, movies and the like)

All these characteristics have their challenges/pathologies as well. A well-known challenge for anticipatory research, for instance, is the so-called Collingridge dilemma (Collingridge 1980): although it is most effective to shape innovative technologies in a societally desirable direction at an early stage of development, it is difficult during this early stage to assess what the societal effects of the technology will be. In more advanced phases societal effects will become clearer, but there is less room for change. In other words, although impacts cannot be easily predicted until the technology is extensively developed and widely used, steering becomes more difficult when the technology has already become entrenched.

Another intricacy involves the interdisciplinarity of ELSA, notably the collaboration between philosophers/bioethicists on the one hand, and STS scholars on the other. The complexities of these collaborations were sometimes underestimated, notably in terms of the various forms of rivalry between these strands of ELSA research, not only over methods, but also over chairs, journals, program committees, funding opportunities and the like. The complexities of academic power play, also *within* ELSA, proved quite fierce on some occasions. But by far the most controversial feature of ELSA, as outlined above, has been ‘proximity’.

Social sciences and humanities experts who were open to the ELSA approach have increasingly found themselves collaborating closely with scientific partners from the life sciences and from technical fields, even in the sense that they were institutionally bound to them. And this has made their work more relevant and well-informed. The question which ELSA communities continuously need to address basically is this: how to combine proximity with intellectual autonomy and societal credibility? The obvious answer is: through dialogue. Rather than undermining independence, proximity to and interaction with life scientists can make ELSA reflections, observations and criticisms more precise, more up-to-date, more targeted, and more relevant. Ideally, the scientists involved do not merely function as objects of research, as is the case in more traditional types of research, but are invited and allowed to comment on (preliminary versions of) ELSA analyses and assessments. Thus, interactive research entails a kind of empirical cycle of its own, where preliminary assessments are developed and tested in the context of critical dialogue and mutual learning. This is not only a benefit in terms of research ethics (granting the ‘other side’ the right to respond), but also makes ELSA analyses and assessments more robust. In the midst of efforts and disputes to address these issues, however, a new approach was suddenly heralded: RRI.

## Part II: the emergence of RRI

### What is RRI? And where does it come from?

The first decade of the 21^st^ century (notably the period 2002 – 2012) were the golden years of the ELSA approach. Fields such as technology assessment, philosophy of science, and bioethics were pushed towards a process of “elsification”, adopting the ELSA profile outlined above (either voluntarily or in order to live up to funding prerequisites). But now, within the EU at least, ELSA as a label seems to have gone out of fashion. The term *Responsible Research and Innovation* is suddenly in vogue. In the Netherlands, for instance, funding for CSG is about to expire while the program “Maatschappelijk Verantwoord Innoveren” (MVI, the Dutch version of RRI) has been launched to fund collaborative projects with the private sector. In call texts issued by this program, run by the Dutch Science Council (NWO),^e^ it is explicitly pointed out that the emphasis should be on furthering socio-economic goals through partnerships with industry and private companies. The program is strongly linked to the Dutch innovation policy, aimed at strengthening ‘top-sectors’ of the national economy through research and innovation. Responsible innovation is *better* innovation, is the general adage, and innovation is expected to strengthen the competitiveness of core Dutch industry. Besides the fact that a rather prominent role is now given to private companies and public-private-partnerships,^f^ this also has consequences for the question which stakeholders are to be seen as the most relevant partners in societal dialogue. The most recent MVI-call (NWO 2012) not only contained a rather restrictive listing of issues that were eligible for funding, but also (for every issue) a pre-fixed list of issues that research proposals were expected to address. Thus the profile of the type of research to be funded was quite explicitly pre-determined, and potential research proposals were steered in fairly specific directions: either acquiesce or perish, as it were.

The question now is: what *is* RRI and in what way does it differ from ELSA? We will argue, first of all, that at first glance RRI is *not* a radical departure from ELSA, and that, in the process of further developing the RRI approach, the inclusion of ELSA’s heritage may well prove essential. Yet, we do see a new emphasis emerging in RRI in comparison with ELSA, namely the focus on socio-economic benefits and collaboration with private and industrial partners.

### ELSA versus RRI?

When it comes to defining (or at least profiling) RRI, it is important to keep in mind first of all that, very much like ELSA, RRI is *not* a (new) discipline or research field. Rather, as we will see, it is a basic strategy to *change* the way in which research and innovation is usually done. And this is one of the aspects which RRI shares with ELSA. Through interactive involvement, RRI initiatives propose to move agenda-setting processes in the direction of societal responsibility. Let us look into this in more detail.

An important voice in advocating RRI is René von Schomberg, a former academic with expertise in philosophy and science and technology studies (see for instance Wheale, von Schomberg, Glasner 1998) who became a key figure at the Governance and Ethics Unit of the European Commission. In the opening lines of *A vision of Responsible Research and Innovation* (2013) he argues that “RRI has become an increasingly important phrase within policy narratives, in particular in Europe, where it will be a cross-cutting issue under the prospective EU Framework Programme for Research and Innovation *Horizon 2020*”. And yet, he emphasises that “there is no agreed definition of the concept, and approaches how it should be implemented may vary”. Indeed, the field is explicitly invited to join the debate as to what RRI (as a top-down signifier) exactly signifies.

In two recent publications on RRI (Schomberg 2011, 2013), the following definition is proposed: *Responsible Research and Innovation is a transparent, interactive process by which societal actors and innovators become mutually responsive to each other with a view to the (ethical) acceptability, sustainability and societal desirability of the innovation process and its marketable products (in order to allow a proper embedding of scientific and technological advances in our society).* As we have seen, interaction between societal actors and innovators has been a key element within the ELSA approach as well. So, what’s new? In a recent interview (2012), pressed to explain the difference between ELSA and RRI more clearly, Von Schomberg argues that RRI sees ethics “as a stimulus, not as an obstacle”. Right at the start of the interview, he is asked whether RRI is really a new approach, or mainly a new label for ELSA? In his reply, Von Schomberg explains the specificity of RRI as follows: “What’s new about RRI is that we no longer see the ethical aspects of new technologies as constraints, as restrictions. Instead, we look at the aims of technology development. Which positive contributions do you wish to obtain from research and innovation? This positive basic attitude is an important difference in comparison with the ELSA approach.” (p. 16) Further on, he argues that RRI rebels against the more traditional approach (which was focussing on the question whether a development has undesired effects), but rather uses the possible positive contributions of (the development of) a technology as an assessment criterion. Moreover, “unlike ELSA, RRI considers the entire innovation process, from research and development to production and distribution” (p. 16).

This downgrading of ELSA to a set of ethical constraints meant to curb negative outcomes and undesired effects of innovation, seems difficult to reconcile with the type of activities that have actually evolved under the ELSA label, as explained above. And similar to RRI, ELSA already had the ambition to become involved in innovation trajectories at a relatively early stage (‘anticipatory’ research). In another paper (Von Schomberg 2011), however, some additional indications concerning the specificity of RRI compared to ELSA are given. Among the typical features of RRI are now mentioned the use of ethics as a *design principle* for technology (for example: privacy through design) as well the ensuring of market accountability through standards, certification, accreditation and labels as a new form of governance to manage the floods of products coming to the market.

From our perspective, the idea that ELSA focusses solely on ‘negative’ consequences and functions basically as a constraint, notably at the ‘end’ stage of the innovation process, is a caricature, clearly at odds with the way ELSA-type research has actually evolved / has functioned in practice. In such cursory comparisons, important key aspects of ELSA, such as anticipation and proximity (see above), are conveniently left out of the picture. Yet, it is clear that some new elements or at least emphases are introduced as well. These are conveyed by the use of terms that are not absent, but relatively rare in ELSA discourse (which has a predilection for studying ‘soft’ rather than ‘hard’ impacts), such as ‘design’, ‘markets’ and ‘accreditation’. It is in this direction, we believe, that the relative newness of the RRI-approach can be found. And that is why the term metonymy is used to specify this shift: there is a clear congruency between the ELSA and the RRI approach, but a number of items that used to be marginal in ELSA are now pushed to a much more prominent position in RRI.

Meanwhile, Von Schomberg’s phrasings reveal much about how science policy in the EU has evolved and is progressing (Blume 1986, Rodrígueza et al. 2013). Today, science is expected to improve the functioning of society by creating innovations. These innovations are needed to maintain affluent living standards in the face of globalization, i.e. the (possible) erosion of the competitiveness of European business, as well as the need to enhance sustainability (not only in an environmental, but also in a socio-economic sense) and other ‘grand challenges’. These innovations are not only to be implemented on the micro-level, so that companies can make a profit and create jobs, but also at the macro-level, leading to system-wide transformations of important industrial sectors and, ultimately, of the European economy as such.

In this sense, it comes as no surprise that Von Schomberg (2013) explicitly links research, and particularly research funding for universities, to tackling the grand challenges facing European societies. These challenges require citizens, researchers and entrepreneurs to channel the change-engine of science towards the common good. For Von Schomberg, the EU Treaty provides the ‘normative anchors’ for this process: sustainable development, competitive social market economy, full employment and social progress, protection and improvement of our environment, and no social exclusion (social justice added to quality of life). Yet, these normative anchors need to be taken as positive triggers for innovation, not as negative constraints. For instance, in the case of possible implementations of these elements one needs to ask what would be the ‘right impacts’, as well as what would be the ‘right processes’ to realise such impacts.

Thus, although some important elements of the RRI approach may sound very much like ELSA, the overall framing is different and linked to innovation and to addressing grand (socio-economic) challenges. In turn, the framing gives much more weight and urgency to the matter of channelling science to the common good. Indeed, the type of innovation needed, and the way these innovations are produced, have (according to Von Schomberg) changed. Nineteenth-century inventor-entrepreneurs created Frankensteinian monsters by themselves, but today, networks of university researchers, companies, pressure groups and individuals may produce innovations (responsible or otherwise) by diffusing accountability, while the consequences can be far more substantial and far-reaching. From a macro-perspective, moreover, society may need forms of innovation which private companies may not bring about by themselves because it is risky and unprofitable (or otherwise makes no sense for them to pursue). Getting a grip on the societal dimensions of innovation - i.e. not just market needs but also the ethical, legal and policy issues involved - could make innovation much more effective. Hence: we need RRI.

### Source 2: options for strengthening RRI

Another source for addressing the question *What is RRI?* is a recent report edited by Jeroen van den Hoven (Chair of the Dutch MVI programme committee) and other prominent voices in the RRI discourse, and published by the European Commission. Again, as we will show, although a close reading reveals a remarkable inability of the authors to flesh out the RRI concept in a precise and convincing manner (notably in contrast to existing approaches), the shift towards innovation and socio-economic development is nonetheless clear.

Initiatives falling under the heading of RRI are bent on (Hoven et al. 2013, p. 12):

- considering societal needs and ethical aspects in research funding programs, e.g. through public and stakeholder dialogue;
- developing criteria for the early appraisal of research and innovation, e.g. technology assessments;
- establishing processes to better integrate societal needs in research and innovation, e.g. trans-disciplinary approaches in sustainability science;
- setting up advisory bodies such as councils on ethical aspects of new technologies.

For anyone familiar with the field, it is obvious that this is more or less exactly what ELSA envisioned to achieve as well. The same applies to the tactics the authors suggest for promoting this type of research through funding strategies:

- “One way to promote RRI is to mainstream RRI in the existing funding programmes. In this case no new funding opportunities on RRI would be allocated, but criteria for RRI would have to be applied across all EU funding programmes. This would not only raise awareness for RRI but also create greater transparency with regard to the provisions for taking into account societal needs and ethical aspects in the proposed research.” (p. 38/39)

And this is how RRI is operationalised by the authors of the report: RRI is anticipatory, inclusive, reflexive and responsive:

- Anticipatory: Anticipation asks researchers and innovators to include new perspectives in the research and innovation process and to think through various possibilities to be able to design socially robust agendas for risk research and risk management.
- Inclusive: Inclusiveness asks researchers and innovators to involve diverse stakeholders (such as users, NGOs, etc.) in the process to broaden and diversify the sources of expertise and perspectives.
- Reflexive: Reflexivity asks researchers and innovators to think about their own ethical, political or social assumptions to enable them to consider their own roles and responsibilities in research and innovation as well as in public dialogue. Reflexivity should raise awareness for the importance of framing issues, problems and the suggested solutions.
- Responsive: If research and innovation claim to be responsible, if it has the capacity to change its direction or shape when it becomes apparent that the current developments do not match societal needs or are ethically contested. Hence, responsiveness refers to the flexibility and capacity to change research and innovation processes according to public values. (p. 58)

Again, this is very much how ELSA has been developed and defined during the past decade.

And yet, the difference is there. In order to see it, however, we must shift the focus of attention from the description of the general methodological profile of RRI (which sounds very much like ELSA) to the broader, socio-economic context. Much like Von Schomberg (2013), the report frames the need for societal involvement and assessment in terms of innovation and competitiveness. Notably, reference is made to “…the ambition of the European Union to ensure that research and innovative ideas can be turned into products and services that create jobs and prosperity, as well as help preserve the environment and meet the societal needs of Europe and the world (p. 11).” The point of RRI is to help achieve this ambition: “RRI has the potential to make research and innovation investments more efficient, while at the same time focusing on global societal challenges.” (p. 16) Inclusion of ethics beforehand, it seems, will lead to less contestation of innovations afterwards.

### So, what is really new?

We end up with a remarkable ‘symptom’: RRI is presented as something new, as a break with the ELSA past, and yet, upon closer inspection, RRI is defined in ways that tend to resemble the ELSA stratagem quite closely. Key proponents of RRI apparently struggle to articulate their own innovation in a convincing way.

This symptomatic failure (*Fehlleistung*) may be seen as a result of the disregard towards (‘repression’ of) the ELSA heritage, followed by the subsequent inevitable ‘return of the repressed’, namely the resurge of recognisable ELSA-speak in the definitions given of RRI. At the same time, however, it points to the fact that the difference is not in the methods or approaches used as such (as is sometimes suggested, notably by stressing the need for anticipation and interaction), but rather in the *finality* of RRI, the overall aim to be achieved, namely: ensuring that the EU economy remains internationally competitive and robust.

Very much like ELSA, RRI builds on existing tendencies in academia and policy to integrate societal research and interaction into science and technology practices. The confusion concerning the question what RRI ‘is’ can only be overcome when, in elaborating the RRI concept, both the continuity (with ELSA) and the discontinuity (the newness of RRI compared to ELSA) is fleshed out with more precision. And the one is a prerequisite for the other, because it is only insofar as the value of ELSA’s heritage is duly acknowledged, that the ‘added value’ of RRI can be articulated with sufficient precision.

In other words, rather than presenting RRI as a complete *novum*, while debunking/ignoring the ELSA experience, we argue that, in terms of proximity, co-creation, stakeholders participation and the like, RRI should build on, rather than break with, the ELSA approach. Proximity and interaction as such are not new (Rodrígueza et al. 2013) and much can be learned from the ELSA heritage on whose accomplishments RRI could be engrafted. Only then can it be pointed out more clearly in what way RRI differs from its predecessor.

It is clear that RRI departs from ELSA most clearly in its focus on economic innovation. RRI is supposed to help research to move from bench to market, in order to create jobs, wealth and well-being. In turn, this focus on innovation has a number of important consequences. Micro-level case studies of knowledge production and innovation processes (so dominant in ELSA) will not be eliminated we expect, but will be complemented by mid- and marco-level studies of transformations and transitions, to bring the broader socio-economic context into view. Substantive normative questions also come into play at this level, particularly the question what kind of society and economy we want (which, of course, includes issues of sustainability and fairness). Private companies will be involved in RRI (being less prominent in ELSA). This will entail a shift of focus from analysing knowledge production to processes of co-design in innovation and public-private interaction.

As a consequence of this, RRI also entails a shift in terms of disciplines. Whereas ELSA was a collaboration between disciplines such as philosophy of science and/or technology, bioethics, STS, CTA, interactive science communication, and the like, RRI requires intense collaboration with a ‘third’ strand of research, namely management and innovation studies, fields that, broadly speaking, investigate how innovations come about, how they are managed and how policy affects them. RRI could profit from including innovation studies as a body of knowledge and/or as a scholarly community, to link the micro- with the macro-level and to make the results of research more relevant for policy makers.

Thus, we expect ELSA to partly merge into RRI, so that *in practice*, RRI will take the viable parts of the ELSA heritage more seriously than is done in printed form. For the ELSA community, RRI may increase the relevance of ELSA, as this type of research may now become part of broader transitory processes. New types of partnerships, notably with industry will evolve, urging the introduction of new research techniques (for instance adopted from innovation studies). However, the ELSA → RRI metonymy also carries a risk: the envisioned merger of the ELSA-agenda (bent on promoting morally justified research) with innovation and industrial agendas may lead to silencing of critical, ‘rogue’ voices and outsiders in the debate, due to increased dependency on private sector parties and policy agendas. In that way, RRI may be faced with an unexpected result: it may be exactly the outsiders, i.e. small firms, small groups of researchers, interdisciplinary projects at the cutting edge of various disciplinary silos, that create the radical innovation we need to transform the EU economy and address the challenges it faces.

### Potential problems with the integration of science and technology under RRI

One important question is *how* an RRI approach could enhance innovation, particularly the beneficial effects of innovation (wealth, jobs, competitiveness, etc.). The RRI label suggests that some innovations will be selected over others, namely the ‘responsible’ ones. This is hard work in any kind of setting, particularly so when it is unclear as to exactly *what* a ‘responsible innovation’ is and *who* will be the one to determine this. It poses tough questions at the micro-level (what to do in the context of a particular RRI project?), at the meso-level (what kind of projects to select and reward?) and at the macro-level (what types of research should we prioritise?). EU funding institutions, as well as the values they represent, are currently targets of dispute (to put it mildly). Which interpretation of responsibility are we to pursue? Relying on ‘process’ and ‘dialogue’ runs the risks of missing the mark and delivering responsible innovations that fail to address the grand challenges on a macro-scale. In the end, RRI revolves around a crucial issue: what kind of innovations and what kind of economy do we want? And who will be the ‘we’?

ELSA researchers (who already ran the risk of *going native* due to their proximity to and involvement with science programs) will (upon entering the promised land of RRI) become increasingly *co-responsible* for the innovations they help to develop. One important aspect of co-responsibility is *promise management*. Promises and expectations have always played an important role in science and technology development. They are currently facing a credibility deficit, a credibility crisis even, due to years of overpromising, i.e. the practice of recycling stereotypical lists of promises time and again for various purposes, until the public sphere becomes saturated with faltered promises and extended time-schedules. A closer involvement of societal stakeholders and future users in the innovation pathways implies that promisory discourse will be opened up to them as well, so that promises become co-constructed as well. At least implicitly, but often explicitly, ‘promise management’ becomes part the projects and activities developed. What promises have been made, to what extent have they been met; has the promised relevance been achieved? This is the handiwork of “promisomics” (Chadwick and Zwart 2013), i.e. the co-constructive framing, monitoring and management of promises, counteracting fabulation and making promisory discourse more convincing, reliable and relevant. How can we frame promises that are reliable, plausible and socially robust? How can we assure that they concord with the needs and expectations of society?

Involving societal input in science and technology innovation implies that RRI- researchers become part of the very processes they study, immersing themselves in research consortia whose work they claim to critically assess. The tension between ‘going native’ and giving voice to critical concerns is there to stay. For the ELSA community, the liaison (or even ‘marriage’ with) RRI will bring up an issue that is uncannily reminiscent of the one that haunted Elsa of Brabant, the female lead in Wagner’s *Lohengrin*: what will be the questions we are no longer supposed to (but will find impossible not to) ask?

## Endnotes

^a^The prominence of the Ethics Review process can be seen from the FP7 web-portal pages on Ethics: http://cordis.europa.eu/fp7/ethics_en.html (last visited 14 May, 2012).

^b^As Krabbenborg (2013) argues, a CTA researcher can move around in different worlds, make observations, ask questions, and point out problematic issues that are more difficult to notice for actors who operate from a particular disciplinary perspective. Notably, ‘bridging events’ can provide an opportunity for actors to freely explore emerging patterns, articulate problematic issues and develop strategies for how to deal with it. Thus, in the context of CTA, the focus shifted from expert reports to early stage preparatory and anticipatory interaction with stakeholders.

^c^The Canadian acronym (GE^3^LS) added the G (for genomics) but dropped the postfix A/I.

^d^Nature, 342 (6246), 9 November 1989, p. 108; doi:10.1038/342108a0.

^e^Ironically, NWO, established in 1950 as ZWO (*Netherlands Organisation of Pure Scientific Research*, with the Z referring to ‘pure’ (i.e. fundamental) research - but this signifier was dropped from the acronym in 1988 in order to indicate the broadening of the mission), was traditionally focussed on funding ‘basic’ rather than ‘applied’ research activities.

^f^Collaboration with industry was (perhaps) less prominent, but far from absent in ELSA, of course, see for instance: Penders and Nelis (2011).
